# Monitoring weed mechanical and chemical damage stress based on chlorophyll fluorescence imaging

**DOI:** 10.3389/fpls.2023.1188981

**Published:** 2023-05-15

**Authors:** Longzhe Quan, Keyong Chen, Tianbao Chen, Hailong Li, Wenchang Li, Tianyu Cheng, Fulin Xia, Zhaoxia Lou, Tianyu Geng, Deng Sun, Wei Jiang

**Affiliations:** ^1^ College of Engineering, Anhui Agricultural University, Hefei, Anhui, China; ^2^ College of Engineering, Northeast Agricultural University, Harbin, China; ^3^ College of Engineering, China Agricultural University, Beijing, China

**Keywords:** weeds, mechanical stress, chemical stress, complex stress, chlorophyll fluorescence

## Abstract

Currently, mechanical and chemical damage is the main way to carry out weed control. The use of chlorophyll fluorescence (CF) technology to nondestructively monitor the stress physiological state of weeds is significant to reveal the damage mechanism of mechanical and chemical stresses as well as complex stresses. Under simulated real field environmental conditions, different species and leaf age weeds (Digitaria sanguinalis 2-5 leaf age, and Erigeron canadensis 5-10 leaf age) were subjected to experimental treatments for 1-7 days, and fluorescence parameters were measured every 24 h using a chlorophyll fluorometer. The aim of this study was to investigate the changes in CF parameters of different species of weeds (Digitaria sanguinalis, Erigeron canadensis) at their different stress sites under chemical, mechanical and their combined stresses. The results showed that when weeds (Digitaria sanguinalis and Erigeron canadensis) were chemically stressed in different parts, their leaf back parts were the most severely stressed after 7 days, with photosynthetic inhibition reaching R=75%. In contrast, mechanical stress differs from its changes, and after a period of its stress, each parameter recovers somewhat after 1 to 2 days of stress, with heavy mechanical stress R=11%. Complex stress had the most significant effect on CF parameters, mainly in the timing and efficiency of changes in Fv/Fm, Fq’/Fm’, ETR, Rfd, NPQ and Y(NO), with R reaching 71%-73% after only 3-4 days of complex stress, and its changes in complex stress were basically consistent with the pattern of changes in its chemical stress. The results of the study will help to understand the effects of mechanical and chemical stresses and combined stresses on CF parameters of weeds and serve as a guide for efficient weed control operations and conducting weed control in the future.

## Introduction

Weeds in agricultural fields have long been one of the major causes of crop yield reduction because they compete directly with crops in consuming resources such as soil nutrients and water (Mézière et al., 2015; Colbach et al., 2019; Sabzi et al., 2020), and weeds are so damaging and vigorous that nearly 10% of crop yield is lost each year due to weed damage alone. Weed control technology is a very important part of agricultural production, and its development can reduce the competition of weeds to crops and improve the efficiency of agricultural production, which is divided into four ways: chemical control, mechanical control, biological control and soil management. Currently, two main methods of abiotic stress, mechanical and chemical, have been used for weed control (Chicouene, 2007; Mulder and Doll, 2017; Merritt et al., 2020; Chang et al., 2021; Qu et al., 2021). Mechanical damage is mainly through external forces that destroy the photosystem II (PSII) donor side of its photosynthetic system (Delaney, 2008; Nishiyama and Murata, 2014), while chemical stresses vary in damage principles and effects depending on the mechanism of action of their herbicides and the site of damage (Harker and O'Sullivan, 2017; Alizade et al., 2021), and compound stresses cause more severe damage (Zhou et al., 2017; Ma et al., 2018). Differences in the location and mode of stress have led to uneven results in weed control. Therefore, different weed damage methods and sites may have different responses, and their damage mechanisms lead to different physiological changes. Accurate monitoring of the phenotypic change information of weeds caused by stress, easy and fast access to weed mortality information, and find the optimal solution for weed control, the lowest cost to reduce weed damage, in order to improve crop yield and quality.

Both destructive and non-destructive methods have been used to detect abiotic stresses and their responses in plants (Gorbe and Calatayud, 2012; Baba et al., 2016). Among these, chlorophyll fluorescence (CF) imaging is one of the most common non-destructive techniques that have been applied to detect abiotic stresses in a range of plants (Dayan and Zaccaro, 2012; Gorbe and Calatayud, 2012; Wang et al., 2018). CF parameters can provide information about the details of mechanical damage and the extent of plant damage due to stress and measure various chlorophyll fluorescence parameters and quenching effects (Harbinson et al., 2012; Li et al., 2017; Schutte et al., 2019; Hassannejad et al., 2020). The use of chlorophyll fluorescence monitoring is based on the theory that plant stress leads to physiological changes in photosynthesis and fluorescence properties of plants (Bolhar-Nordenkampf et al., 1989; He et al., 2018). Snel et al. (1998) assessed the effect of the herbicide linuron on photosynthesis in freshwater algae by using chlorophyll fluorescence to measure the efficiency of photosystem II electron flow (ETR) (Snel et al., 1998). Raji et al. (2016) studied the changes in photosynthetic parameters such as net photosynthetic rate (Pn) and stomatal conductance (gs) in plants such as taro and sweet potato using fluorescence under herbicide and drought stress (Raji et al., 2016). Agostinetto et al. (2016) showed that photosynthetic rate, chlorophyll content, stomatal conductance and transpiration rate of wheat plants were reduced under the herbicides metribuzin, metsulfuron and 2,4-D stresses (Agostinetto et al., 2016). The above many studies show that chlorophyll fluorescence technique can be a good way to monitor the physiological changes of green plants when they are subjected to chemical and other stresses.

Many scholars have used chlorophyll fluorescence imaging techniques to study the effects of mechanical and chemical stresses on weeds, such as herbicides, machinery, high temperature, salinity, and drought (Hogewoning and Harbinson, 2007; Shin et al., 2020b; Lazarevic et al., 2021). Fuks et al. (1992) studied the photosynthetic performance of weeds using fluorescence of the oxidized PSII primary quinone receptor QA to determine if alterations in the 32-kD protein of photosystem PSII altered resistance to triazine herbicides (Fuks et al., 1992). Under herbicide stress, the chlorophyll content and photosynthetic capacity of weeds were reduced and the photochemical composition of the measured fluorescence quenching was altered. Linn et al. (2020) used chlorophyll fluorometry to measure weeds in the field after herbicide treatment and assessment of herbicide sensitivity (Linn et al., 2020) and determined that the fluorescence parameter Fv/Fm could be a strong indicator for evaluating weed sensitivity to herbicides. Fedotov et al. (2016) used fluorescence spectroscopy to monitor the effects of stress caused by mechanical damage in turfgrass and showed that the fluorescence ratio can be considered a reliable feature of plant stress status (Fedotov et al., 2016). Mechanical damage causes damage to the donor side of PSII, resulting in the production of large amounts of reactive oxygen species (ROS) in plants such as weeds, and excessive accumulation of ROS in plants (Choudhury et al., 2016), induces aldehyde formation and increases photosynthetic efficiency in response to stress by increasing the number of reaction centers per unit area (Wojtaszek, 1997; Maresca et al., 2020). These studies highlight the potential and effectiveness of using chlorophyll fluorescence for monitoring weeds subjected to herbicide versus mechanical stress.

At present, many researches are based on one factor of weed damage as the evaluation of weed control standard, but weeds are not affected by a single factor when they are under stress, such as species, damage mode and degree, damage site and application dose, etc. Considering many factors and observing the change pattern after stress, it is significant to reveal the damage mechanism of mechanical and chemical stress, and also can provide a strong theoretical basis for the related weed control technology. Therefore, the aim of this study was to investigate the changes in CF parameters and physiological response patterns of different species of weeds (Digitaria sanguinalis and Erigeron canadensis) at their different stress sites and levels under chemical, mechanical and their combined stresses.

## Materials and methods

### Plant material and damage conditions

Through the preliminary review of data and actual research, this study selected typical weeds in East China, where crops are biannual, tillage is frequent, and there are many weed species, making prevention and control difficult. Therefore, according to the morphological classification of weeds, the experiments selected the most representative weeds of the region, Erigeron canadensis (Broadleaf family) and Digitaria sanguinalis (Gramineae family), as the sample weeds to carry out relevant studies (Chism and Bingham, 2017; Kelly and Coats, 2017; He et al., 2023). Maize is the main food crop in the region, and weed control in maize farmland is especially important, whose stalk weed control period is mostly the 3-5 leaf stage of maize. During this period, the field is mostly 5-10 leaves age and the plant height is mostly 2-5 cm; Digitaria sanguinalis is mostly 2-5 leaves age and the plant height is mostly 1-3 cm, so we only collected weeds randomly before the 5 leaves stage of maize.

**Figure 1:**
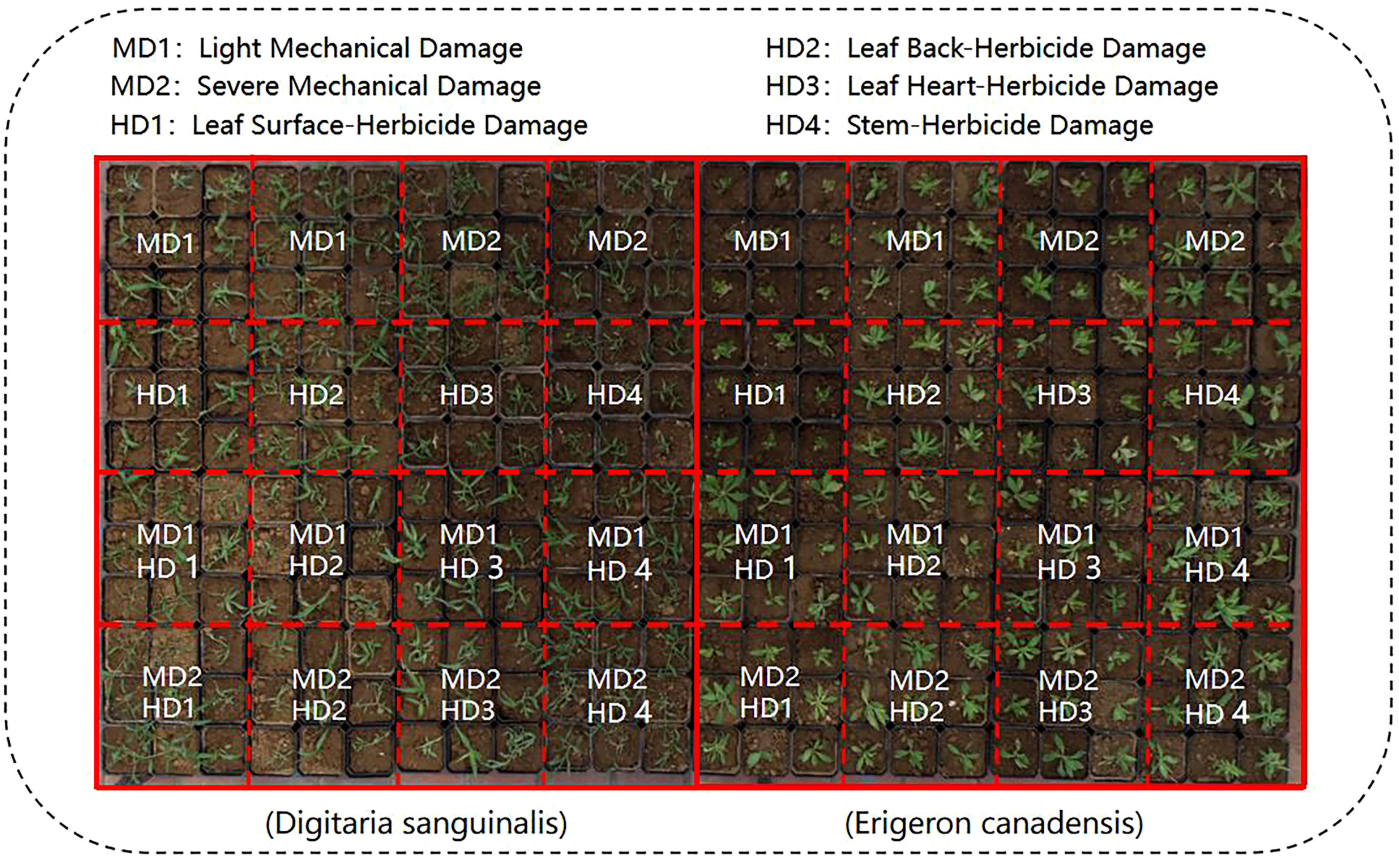
Visible visual appearance of the two weed samples collected.

In this experiment, wild weeds were transplanted into individual standard square pots (10cmx10cmx8cm) in the wild using a field transplanting method (Hazrati et al., 2016), and total transplanting about 300 plants. The transplanted weeds were placed in a shaded position for slow transition, and then the weeds were placed in daylight conditions to simulate the real field natural environment, and the weed samples are shown in (**Figure 1**). Treatment divisions included leaf surface chemical stress HD1; leaf back chemical stress HD2; leaf heart chemical stress HD3; stem chemical stress HD4; light mechanical stress MD1; severe mechanical stress MD2; light mechanical complex stress MD1-HD; and severe mechanical complex stress MD2-HD.

### Preparation of stress samples and fluorescence image acquisition

**Figure 2:**
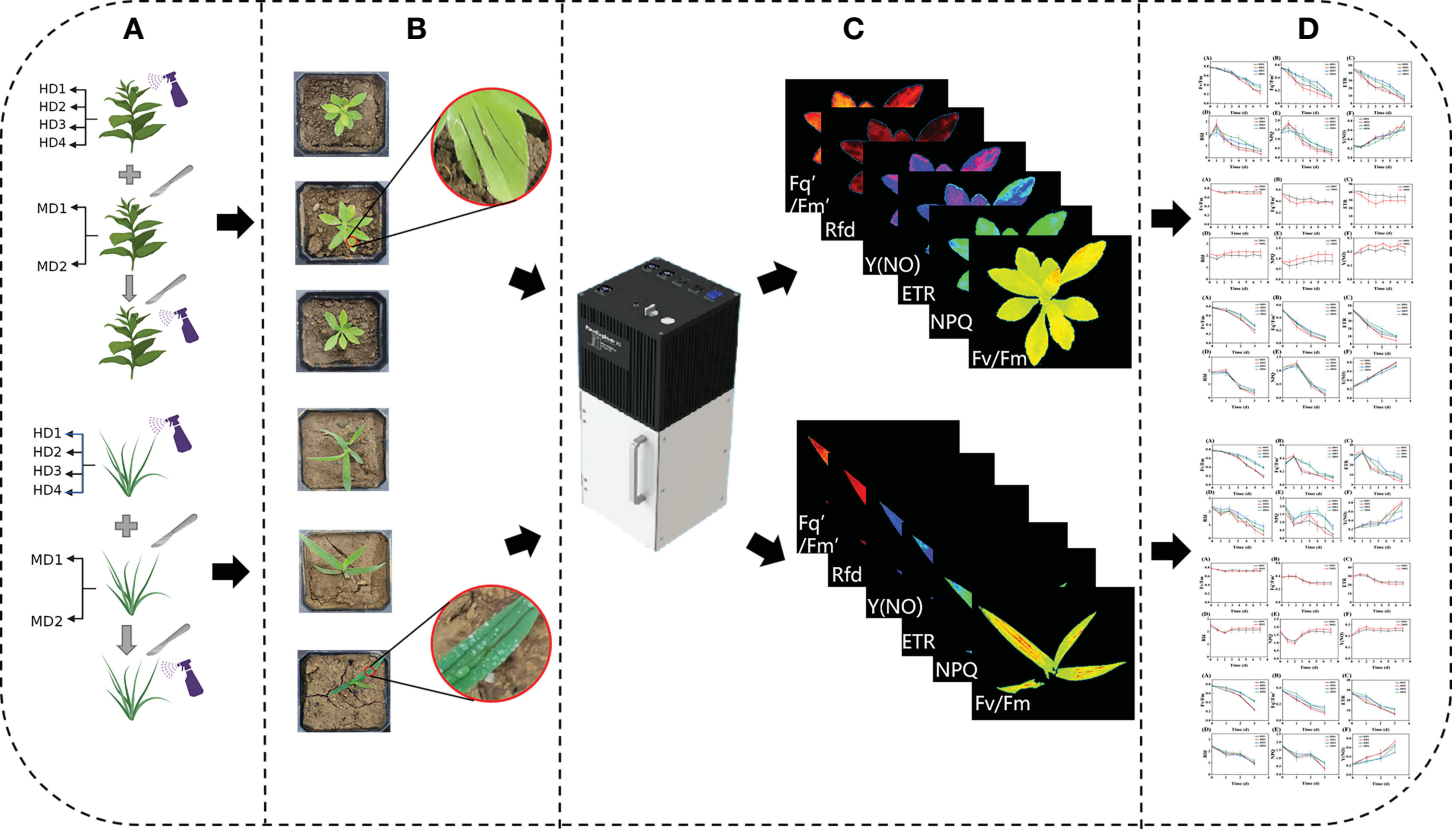
Experimental processing flow chart, **(A)** shows different stress treatments; **(B)** RGB images of sample weeds after treatment; **(C)** acquisition of fluorescence images; **(D)** data analysis.

Weed plants in good growth condition under daylight conditions will be damaged under natural environment after a slowing period of days. Prior to damage experiments on weeds, a chlorophyll fluorescence imager was used for predictive quantities, and for chlorophyll fluorescence imaging, a mobile chlorophyll fluorescence imaging system, PlantExplorer^XS^ (Pheno Vation, China), which is a mobile chlorophyll fluorescence measurement system specifically designed for field, greenhouse, climate chamber, and laboratory scenarios that can be moved, and set the average pixel value<0.75 for Fv/Fm images of weed plants were screened out to ensure that the experimental weeds were healthy weed plants before they underwent damage (Dong et al., 2020). At present, precision-to-rake application technology has become a trend in weed chemical control (Quan et al., 2022), and for its needs, herbicides were selected from the widely used and broad-spectrum tactile inactivating glufosinate (C_5_H_15_N_2_O_4_P) (Krausz et al., 2017; Tharp et al., 2017; Takano and Dayan, 2020), which has a wide herbicidal spectrum, low toxicity, high activity and good environmental compatibility. The general difference in chemical damage is mostly a distinction of site or dose (Tharp et al., 2017; Ali et al., 2020), so the dose used in the study was proportioned according to the actual local spraying situation, and the recommended dose was about 2500 g a.i./hm^2^, the dosage of single plant agent is 10ml and applied to different parts of the leaf surface, leaf back, leaf heart and stem of the weed respectively (**Figures 2A, B**) (Mehmood et al., 2018). The difference in mechanical damage is mostly the distinction of damage mode and degree and the damage area is generally stem and leaf, so the mechanical damage was uniformly done by scratching the leaf with a blade to simulate the damage caused by field weeds due to weeding machinery operations, the degree of damage is divided by the area of the damaged leaf area and the number of scratches, the damage area ≤ 30% and the number of damaged leaf scratches for 1-2 for light damage, damage area ≥ 50% and the number of damaged leaf scratches for 3-4 for severe damage (**Figures 2A, B**) (Vitta and Quintanilla, 2017), complex stress is treated by simultaneous mechanical and chemical stresses. The experiment was divided into two main parts: a single-factor mechanical or chemical damage stress test and a compound factor mechanical and chemical damage stress test.

Chlorophyll fluorescence images were collected at 9:00 a.m. daily, and the damage treatment was performed in groups of three plants on the same site, averaged, monitored for 7 days and cycled (**Figure 2C**) and analyzed (**Figure 2D**). The Fv/Fm parameter is the most visual representation of the changes in photosynthetic capacity of plants, and the obtained Fv/Fm values were used to calculate their photosynthetic inhibition rate according to the following equation


$$R=\frac{\left(N-T\right)}{\left(N\right)}\times 100\%$$

*R*——Photosynthetic inhibition rate;
*T——*The average photosynthesis of weeds after duress;
*N*——The average photosynthesis of control weeds;

### Selection of chlorophyll fluorescence parameters

For the selection of chlorophyll fluorescence parameters, CF parameters on the upper surface of all leaves of intact weed plants were obtained after measurement using chlorophyll fluorescence imaging, said CF parameters were measured independently after every 24 h at the onset of stress, fluorescence collection yielded a total of 14 fluorescence parameters, in this study, Fv/Fm, Fq’/Fm’, ETR, Rfd, NPQ, and Y(NO) (Perez-Bueno et al., 2019; Ali et al., 2020; Shin et al., 2020a; Wang et al., 2021). And these parameters can clearly express the information about the changes in the photosynthetic system when the weed is stressed, the details of the six CF parameters evaluated are shown in (**Table 1**).

**Table 1:** The chlorophyll fluorescence parameters used in this study.

Parameter	Formula	Description
Fv/Fm	(Fm - Fo)/Fm	Maximum quantum yield of PSII photochemistry measured in the dark-adapted state
Fq’/Fm’	(F’m - F’o)/F’m	Exciton transfer efficiency from antenna pigments to the reaction center of photosystem II in the light-adapted state
ETR	Fm− (Fm× PPFD × 0.5)	Electronic transmission rate in PSII
NPQ	(Fm - F’m)/F’m	Non photochemical quenching of maximum fluorescence
Rfd	(Fm - Fs)/Fs	Ratio of fluorescence decline
Y(NO)	1/[NPQ+ 1 + qL(Fm/Fo -1)]	Quantum yield of non-regulated energy dissipation in PSII

### Statistical analysis

The results of CF fluorescence parameters were averaged over 3 biological replicate experiments. Statistical analysis was performed using SPSS software (Ver. 20; SPSS). Statistical differences between the means of the two groups were analyzed using analysis of variance (ANOVA) and Duncan’s multiple polar difference test, P<0.05. The treatment protocols and their interactions were analyzed using a mixed model one-way ANOVA, P<0.05. The effects of treatments included (leaf surface chemical stress, HD1; leaf back chemical stress, HD2; leaf heart chemical stress, HD3; stem chemical stress, HD4; light mechanical stress, MD1; severe mechanical stress, MD2; light mechanical complex stress MD1-HD; severe mechanical complex stress MD2-HD), and time (0d-7d; for repeated measurements).

## Results

**Figure 3:**
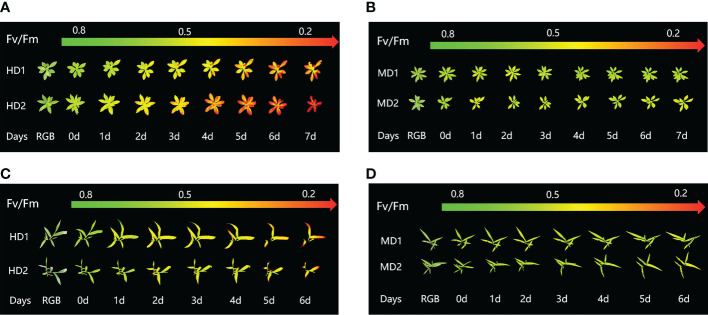
Fluorescence images of Fv/Fm parameters were captured daily during the experimental period, and the changes of Fv/Fm parameters images of Erigeron canadensis and Digitaria sanguinalis under different parts of Erigeron canadensis and Digitaria sanguinalis with different stresses were monitored continuously. **(A, B)** shows the image changes of Fv/Fm parameters in different parts of Erigeron canadensis with different levels of mechanical stress, and **(C, D)** shows the image changes of Fv/Fm parameters in different parts of Digitaria sanguinalis with different levels of mechanical stress.

Chlorophyll fluorescence imaging is a new technique for plant phenotyping that has been used to study the physiological and morphological response of weeds to chemical and mechanical stresses. Phenotypic characteristics were assessed at the onset of stress treatment (0d) and at the longest continuous time of weed decay (1d to 7d) after treatment onset. Pseudo-color images showing the effects of chemical, mechanical and combined stress on selected parameters Fv/Fm are shown in (**Figure 3**).

### Effect of chemical stress on different parts of two weeds on CF parameters

**Figure 4:**
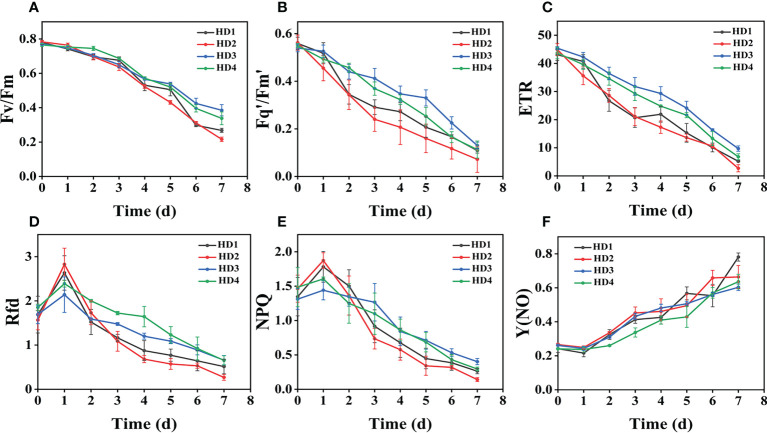
Fluorescence curves of each parameter of CF obtained by capturing each day during the experiment, **(A–F)** shows the variation of each CF parameter of Erigeron canadensis with treatment time under chemical stress treatment at different sites. Each plot point represents the mean ± SD of three biological replicate determinations. Refer (**Table 1**) for the description of each parameter.

**Figure 5:**
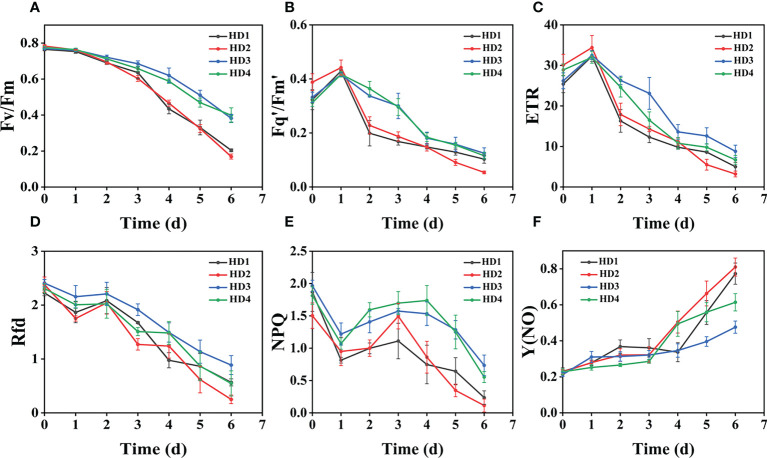
Fluorescence curves of each parameter of CF obtained by capturing each day during the experiment, **(A–F)** shows the variation of each CF parameter of Digitaria sanguinalis with treatment time under chemical stress treatment at different sites. Each plot point represents the mean ± SD of three biological replicate determinations. Refer (**Table 1**) for the description of each parameter.

The images of the effects of chemical stress treatment on Fv/Fm in Erigeron canadensis and Digitaria sanguinalis after 0-7 days are shown in (**Figures 3A–D**). From the site of chemical stress effect, the most obvious degree of change was monitored under chemical stress treatment in the leaf surface (HD1) and leaf back (HD2), and the back of the leaf was the most rapidly affected by chemical stress in comparison with the two. All CF parameters, except Y(NO), decreased during herbicide stress at different sites, with the most pronounced decreasing trend under leaf back treatment (**Figures 4A**, **5A**). The most significant stress changes were obtained from Fv/Fm monitoring during 3 to 4 days under chemical stress treatment in Erigeron canadensis and Digitaria sanguinalis, and the direct cause of the decrease in photosynthetic rate was related to the mechanism of action of glufosinate (Richardson et al., 2007). Fv/Fm, the maximum quantum yield of PSII, the Fv/Fm parameters of Erigeron canadensis and Digitaria sanguinalis decreased insignificantly from day 0 to 1. In (**Figure 4A**), Fv/Fm changed significantly in different parts of the treatment from day 3, and the stress decrease in the back part of the leaf changed significantly, and the most obvious Fv/Fm value in the back part of the leaf decreased less than 0.2 after 7 days, and the photosynthetic inhibition rate R=75%. Fq′/Fm′, which is the effective quantum yield of PSII, (**Figures 4A**, **5A**) also decreased after different site stress treatments, and the trend of change was distinguished clearly, with a higher decrease than that of Fv/Fm. ETR, the electron transport rate of chlorophyll fluorescence in PSII, showed a similar trend to Fq’/Fm’ in different sites experimentally treated for stress, with Fq’/Fm’ and ETR showing a significant increase from 0 to 1 day after treatment, decreasing fastest from 1 to 2 days, and becoming relatively slow after 2 days. Rfd, an important parameter indicating the attenuation of chlorophyll fluorescence, also showed similar trends to NPQ on different sites of experimental treatments of stress. NPQ is a very important non-chemical quenching parameter, and in (**Figure 4E**) Erigeron canadensis NPQ shows a transient increase during 0-1 days at the beginning of the stress. it shows a gradual decrease during 1-7 days, especially the stress change is most obvious in the back part of the leaf treatment, it is interesting to note that in Digitaria sanguinalis the changes in Rfd, NPQ from day 0-1 are reversed. Y(NO) indicates the components of the effectiveness of the photoprotective mechanism, and all other non-photoprotective components except heat diffusion, which are shown in (**Figures 4F**, **5F**) to decrease to a certain extent in 0 to 1 day after stress treatment in different parts of Erigeron canadensis and Digitaria sanguinalis, and show a significant change of increase in the subsequent time, The results showed significant differences in all CF parameters.

### Effect of different levels of mechanical damage acting on two weeds on CF parameters

**Figure 6:**
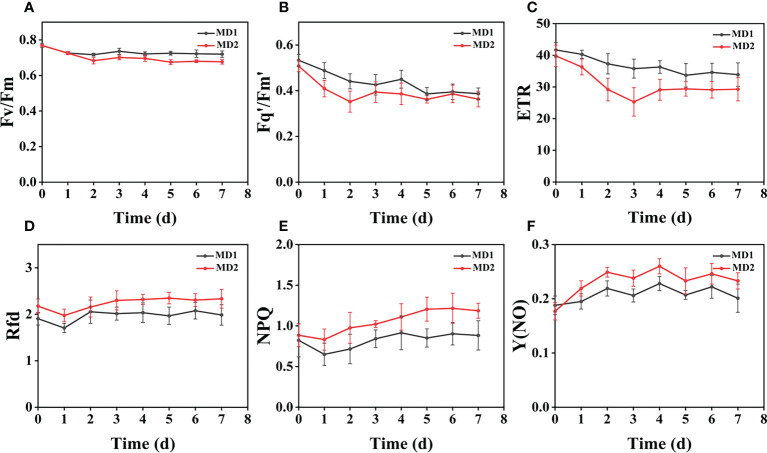
The fluorescence curves of each CF parameter were obtained by capturing each day during the experiment, and **(A–F)** are the changes of each CF parameter of Erigeron canadensis with treatment time under different levels of mechanical stress treatment. Each plot point represents the mean ± SD of three biological replicate determinations. Refer (**Table 1**) for the description of each parameter.

**Figure 7:**
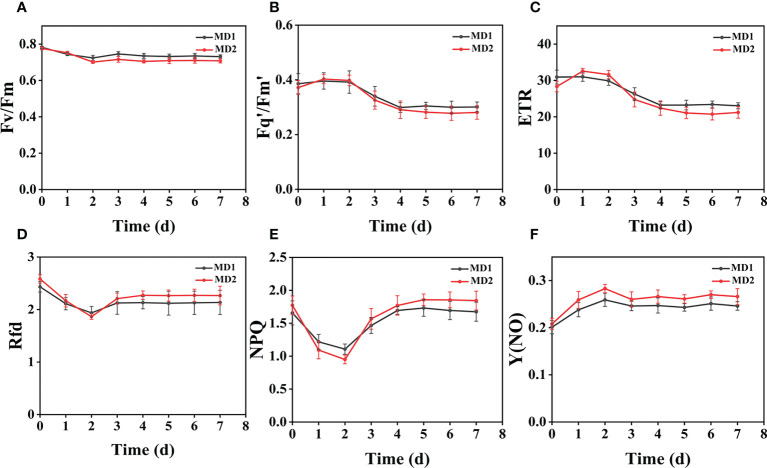
The fluorescence curves of each CF parameter were obtained by capturing each day during the experiment, and **(A–F)** are the changes of each CF parameter of Digitaria sanguinalis with treatment time under different levels of mechanical stress treatment. Each plot point represents the mean ± SD of three biological replicate determinations. Refer (**Table 1**) for the description of each parameter.

From (**Figures 3B, D**), we can see the image changes of the effect on Fv/Fm between Erigeron canadensis and Digitaria sanguinalis after 0-7 days of mild (MD1) and severe (MD2) mechanical damage treatments. The CF parameters in (**Figures 6A–F**, **7A–F**) were influenced by different degrees of mechanical damage. Among the light and heavy mechanical damage conditions, heavy mechanical damage had the greatest effect on all CF parameters. Among all CF parameters, the variation of Erigeron canadensis and Digitaria sanguinalis parameters were basically the same except for Fq’/Fm’ and ETR. In (**Figures 6A**, **7A**), Fv/Fm showed a decreasing change from 0 to 2 days, and a slow increase from 2 to 7 days, and a more stable fluctuation in a certain range, and R=11% for severe mechanical stress. The changes observed in Fq’/Fm’ and ETR during mechanical stress in (**Figure 6**) show similar trends to the changes in Fv/Fm, and the results after stabilization were somewhat lower compared to the initial ones, with the difference that ETR showed a decreasing trend from 0 to 3 days and lasted longer compared to Fv/Fm. The changes of NPQ and Rfd parameters are approximately the same, both decrease rapidly from 0 to 1 day due to mechanical damage, slowly return to stability from 1 to 7 days, and the stable results are elevated compared to the initial ones. Y(NO) in (**Figures 6F**, **7F**) increases when subjected to mechanical damage and stabilizes within a certain range of fluctuation after 2 days after the damage.

### Effect of compound damage stress on CF parameters by acting on different parts of two weeds

**Figure 8:**
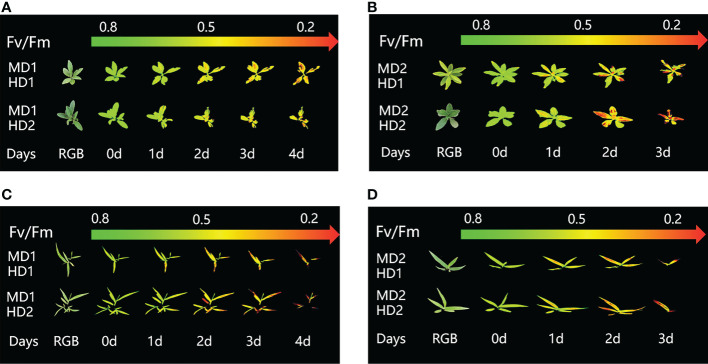
Fluorescence images of Fv/Fm parameters were captured daily during the experimental period, and the changes of Fv/Fm parameter images of Erigeron canadensis and Digitaria sanguinalis under the compound stress mode of chemical damage and different mechanical degree of damage at different parts of Erigeron canadensis and Digitaria sanguinalis during the continuous monitoring period. **(A, B)** shows the image changes of Fv/Fm parameters for light and heavy mechanical compound stresses in Erigeron canadensis, and **(C, D)** shows the image changes of Fv/Fm parameters for light and heavy mechanical compound stresses in Digitaria sanguinalis.

**Figure 9:**
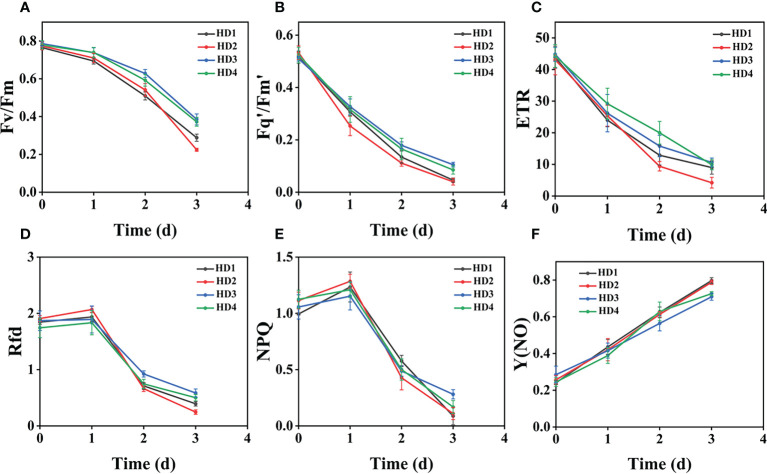
Fluorescence curves of each parameter of CF obtained by capturing each day during the experiment, **(A–F)** are the changes of each CF parameter of Erigeron canadensis with treatment time under f heavy mechanical complex stress treatment. Each plot point represents the mean ± SD of three biological replicate determinations. Refer (**Table 1**) for the description of each parameter.

**Figure 10:**
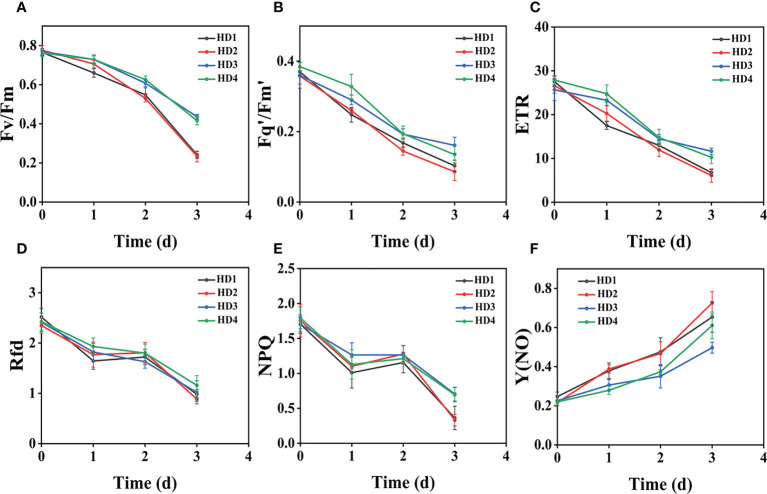
Fluorescence curves of each parameter of CF obtained by capturing each day during the experiment, **(A–F)** are the changes of each CF parameter of Digitaria sanguinalis with treatment time under f heavy mechanical complex stress treatment. Each plot point represents the mean ± SD of three biological replicate determinations. Refer (**Table 1**) for the description of each parameter.

(**Figures 8A–D**) shows the image changes of the effect of compound damage stress on Fv/Fm after the treatment of four different parts of light and heavy mechanical and chemical stresses, and the more severe the compound stress, the more obvious the changes of spatial heterogeneity (**Figures 8B, D**). The weed mortality status resulting from 3 days of heavy mechanical complex stress and 4 days of light mechanical complex stress in (**Figures 8A, C**) is the same and the change pattern is almost the same (**Figures 9A**, **10A**). The most significant changes in Fv/Fm for both light and heavy mechanical complex stresses of Erigeron canadensis and Digitaria sanguinalis were observed after 2 days, and Fv/Fm reached the status of chemical stress treatment for 7 days with R=71%-73% at 3-4 days of complex stress treatment. The changes of Fq’/Fm’ and ETR in (**Figures 9B, C**) are particularly pronounced from 0 to 1 day after the composite stress broke the cuticle hindrance, and the changes of Fq’/Fm’ and ETR in (**Figures 10B, C**) in Digitaria sanguinalis also decreased from 0 to 1 day compared with the corresponding parameters of chemical stress in (**Figures 5B, C**). Erigeron canadensis and Digitaria sanguinalis Rfd and NPQ showed significant changes in compound stress than single stress, and the compound stress Y(NO) did not show a decreasing trend in 0-1 days of treatment, unlike the changes in chemical stress.

## Discussion

### Effect of chemical stress on different parts of two weeds on CF parameters

Chemical stress is a very important abiotic stress that will have a good effect on weed growth control and extermination, but the same application to different parts of the weed performance is also different. From (**Figures 3A, C**) leaf back (HD2), it can be observed that there is a change in area when the treatment proceeds to day 5-6, which is due to the cotyledons having a surface representation of stress, cotyledons wilting, chlorophyll disappearing, and Digitaria sanguinalis approaching a state of death more quickly than Erigeron canadensis when subjected to chemical stress, probably because of the very dense tomentum growing on the leaves of Erigeron canadensis, making it less accessible to the drug solution (Yu et al., 2009). The Fv/Fm parameters of Erigeron canadensis and Digitaria sanguinalis decreased insignificantly from 0 to 1 day, probably due to the obstruction of cuticle (Richardson et al., 2007; Kamtsikakis et al., 2021). The decrease in Fv/Fm values with time in this experiment indicates that photoinhibition of leaves occurred under herbicide glufosinate stress. Fv/Fm in (**Figure 4A**) changed significantly from day 3 onwards for different parts of the treatment, with significant changes in the decline of stress in the back part of the leaf, but in actual production, spraying the foliage is easier to achieve than the back part of the leaf (**Figures 5B, C**). Variation of Fq’/Fm’ and ETR parameters of Digitaria sanguinalis with respect to those corresponding to Erigeron canadensis in (**Figures 4B, C**). The Fq’/Fm’ of Erigeron canadensis and Digitaria sanguinalis were the operational efficiency of PSII photochemistry, while the ETR parameters were also related to photochemistry, and the changes of both were different, probably due to different weed species and different sensitivity to glufosinate.

The variation of Rfd is closely related to the non-photochemical quenching of NPQ, which explains why the variation of Rfd is similar to that of NPQ. In (**Figures 4D, E**) Rfd and NPQ, however, showed a decreasing and then increasing trend from 0 to 2 days, which showed opposite changes to the parameters corresponding to the Erigeron canadensis. The trend of rising and then decreasing NPQ in Erigeron canadensis is due to the generation of photoinhibition (Lu et al., 2003). The rising trend of NPQ in the early stage indicates that the excess light energy absorbed by the antenna pigments in the PSII center is consumed by initiating the NPQ pathway to protect the activity of photosynthetic organs from damage or reduce the degree of damage, which likewise reflects that non-photochemical quenching is an important mechanism that can protect photosynthesis from proceeding smoothly. However, the NPQ tends to decrease with more severe chemical stress, which may be due to the blockage of the thermal dissipation mechanism due to severe chemical stress to the extent that excess light energy cannot be efficiently dissipated through non-photochemical quenching pathways (Chmeliov et al., 2019; Wilson and Ruban, 2020), the reduced heat dissipation capacity, the limitation of CO_2_ assimilation and the imbalance of photochemical activity in photosystem II are factors that lead to energy overexcitation and subsequent photoinhibition (Song et al., 2015; Vodeneev et al., 2018; Chmeliov et al., 2019). The changes in NPQ in the pre-Digitaria sanguinalis period were the opposite of those in Erigeron canadensis, which may be due to insufficient light energy absorption by the antenna pigments in the PSII center, or may be due to the different stress responses to chemical stress caused by different weed species. The increase in Y(NO) levels under chemical stress conditions implies that the weeds were subjected to extreme stress during the experiment, indicating that the leaves developed invisible drug damage such as impaired photosynthetic electron transfer and reduced reaction center activity under herbicide stress, and that NPQ was also reduced under extreme stress conditions (Brugger et al., 2017; Tietz et al., 2017). Overall, chemical stress is highly destructive to the photosynthetic system of weeds, with significant effects on all CF parameters.

### Effect of different levels of mechanical damage on CF parameters of two weeds

Mechanical damage is one of the most common forms of stress in weeds and induces multiple response mechanisms, which are also reflected in chlorophyll fluorescence when weeds are subjected to mechanical stress, and all parameters monitored respond when subjected to mechanical stress. The greater the degree of mechanical damage, the more pronounced the degree of change in weed fluorescence, and mechanical stress can damage weed leaves, possibly due to damage to chloroplast membranes and structures, increased chlorophyllase activity, and excessive accumulation of reactive oxygen species (ROS) due to weed resistance production after stress (Mhamdi and Van Breusegem, 2018; Mittler et al., 2022; Yan et al., 2023). It affects the photosynthesis of the weed for a period of time, leading to the impairment of the PSII community side, so that photosynthetic electron transfer is affected, which also explains the decrease of Fv/Fm, Fq’/Fm’, and ETR in Erigeron canadensis from 0 to 3 days before mechanical stress.

Light and heavy mechanical damage can have different effects on the change of Fq’/Fm’ and ETR in Digitaria sanguinalis, and in (**Figures 3B, D**) it can be observed that there is a significant change in color for heavy mechanical damage (MD2). In (**Figures 7B, C**), the Fq’/Fm’ and ETR of Digitaria sanguinalis first increased and then decreased, which may indicate that the stress response of Digitaria sanguinalis leaves to compensate for the lack of light energy absorption due to damaged antenna pigments by increasing the efficiency of the PSII active reaction center while mechanical damage stress occurred (Appenroth et al., 2001). The different changes in Fq’/Fm’ and ETR between Erigeron canadensis and Digitaria sanguinalis may be due to the different weed species and sensitivity to glufosinate, and also indicate that Erigeron canadensis are more sensitive to glufosinate and glufosinate is more effective on Erigeron canadensis (Steckel et al., 2017). The stabilized values were slightly lower than the initial ones, indicating that mechanical damage stress caused damage or inhibition of antennal pigments, resulting in a reduction of energy absorbed by antennal pigments and energy captured by reaction centers, and that this damage was permanent.

Rfd and NPQ parameters increased photosynthetic efficiency and decreased light energy consumption by non-photochemical quenching when subjected to mechanical stress, which explains why Rfd and NPQ decreased from 0 to 1 day. Since mechanical damage was always present, photosynthetic rate decreased with time and thermal consumption of light energy through non-photochemical quenching pathway increased, so Rfd and NPQ increased but did not change significantly in the subsequent period. Y(NO) increased significantly from 0 to 2 days, indicating that the weed was under the most severe stress during this period. The mechanical damage damaged the PSII community side, resulting in photochemical energy conversion and protective regulatory mechanisms (such as thermal diffusion) were not sufficient to completely consume the light energy absorbed by the weed, and after 2 days, the photosynthesis and physiology of the Erigeron canadensis gradually recovered to reach equilibrium and remained relatively stable, so Y(NO) also tended to stabilize. In conclusion, mechanical stress will destroy the internal physiological structure of weeds and affect the normal work of photosynthetic system, and each CF parameter will fluctuate, but after 2 days each CF parameter will have some recovery.

### Comparative analysis of the effects of mechanical and chemical stresses on CF parameters of two weeds

The most obvious difference between chemical stress and mechanical stress is that chemical stress is irreversible in killing weeds. Regardless of the location where chemical stress acts, chemical stress affects the weed’s individual life by disrupting its physiological patterns and preventing its growth. In contrast, mechanical stress limits weeds by the degree of damage to weeds by weeding machinery, which may be able to stop weed growth to a certain extent, but when the degree of mechanical damage is not sufficient, weeds will still come back. The changes of the two stresses were more obvious by fluorescence parameters, and the R=75% in the back part of the leaf of chemical stress was much greater than the R=11% of heavy mechanical stress.

Fv/Fm responded to the potential maximum photosynthetic capacity of the weed, and the persistent and significant decrease in Fv/Fm of chemically stressed weeds indicated that the damage to the photosynthetic capacity of the weed by chemical stress was irreversible. The degree of variation in the corresponding Fq’/Fm’, ETR, Rfd, and NPQ parameters all responded to the greater damage to the weed by chemical stress, and weed mechanical and chemical stresses behaved differently in the 0-1 day stress response to these four parameters, with chemical stress stresses being obvious, while the stresses exhibited by mechanical stresses were not. This may be due to the mechanical stress directly damage the physiological structure of the leaf, the damage is serious and rapid, fluorescence interval of 24 h monitoring, may be the next monitoring, mechanical stress stress response to manifest the effect has passed, resulting in fluorescence monitoring cannot be monitored or monitoring the effect is not obvious (Liu et al., 2019; Shetty et al., 2019; Li et al., 2020). The continuous increase in the Y(NO) parameter is an indication that the weed is under extreme stress. In contrast to the changes in the fluorescence parameters of mechanical stress, the weeds only changed significantly in the initial 1-2 days of stress, and the parameters hardly changed after the physiological changes of the weeds stabilized.

### Effect of complex damage stress on CF parameters by acting on different parts of two weeds

(**Figures 8A–D**) shows the image changes of the effect of compound damage stress on Fv/Fm after the treatment of four different sites of light and heavy mechanical and chemical stress, the underlying cause of spatial heterogeneity of weeds on CF images is leaf loss of greenness, due to the accelerated degradation of chlorophyll and proteins after weeds are subjected to compound stress, the degradation of biomolecules in plants leads to the release of ROS, and ROS on The oxidative damage produced by the plant includes the accelerated production of ethylene, the main hormone that induces cellular senescence, which leads to the yellowing of leaves (Dubois et al., 2018; Katayose et al., 2021).

Fv/Fm in (**Figure 9A**) and (**Figure 10A**) reached the status of chemical stress treatment in (**Figure 4A**) and (**Figure 5A**) for 7 days with R=71%~73% at 3 days of compound stress treatment, indicating that mechanical stress accelerated the rate of action of chemical stress after breaking this obstructive layer of weed leaf cuticle and greatly accelerated the time of weed death (**Figure 9A**). The most significant changes in Fv/Fm were observed after 2 days for heavy mechanical complex stresses of Erigeron canadensis and Digitaria sanguinalis, which means that although mechanical damage broke the physical defense of leaves, only a small portion of glufosinate acted directly on cells, and most of it still needed to be transported in xylem to act on cells, inhibiting glutamine (GS) production and reducing photosynthetic yield, a process that takes time (**Figures 9B, C**). The changes in Fq’/Fm’ and ETR of Erigeron canadensis were especially obvious from 0 to 1 days after the composite stress broke the cuticle hindrance, which was due to the inhibition of photosynthesis by the direct entry of glufosinate into the leaf cells where the action occurred, reducing the efficiency of PSII photochemical operation and the photosynthetic electron transfer (**Figures 10B, C**). Digitaria sanguinalis Fq’/Fm’, ETR compared to the corresponding parameter changes of chemical stress in (**Figures 5B, C**) at 0 to 1 day also decreased, this is because the stress response of the weed will be somewhat to resist the stress when it is suddenly damaged by such a large stress, due to the extreme, permanent and irreversible damage caused by the complex stress to the internal physiology and structure of the weed, the physiological defense mechanism of the weed is far from sufficient to resist the damage of the complex stress (**Figures 9D–F**) is similar to the variation of (**Figures 4D–F**) and (**Figures 10D–F**) is similar to the variation of (Figure 5D-F), which may be due to the fact that their corresponding species are the same. Erigeron canadensis and Digitaria sanguinalis Rfd and NPQ showed significant changes under compound stress than single stress, suggesting that Erigeron canadensis and Digitaria sanguinalis were subjected to severe stress resulting in imbalance of PSII photochemical activity, energy overexcitation and photoinhibition, and leading to photooxidative damage with high production of reactive oxygen species (ROS) in chloroplasts (Ivanov and Khorobrykh, 2003; Ksas et al., 2015). Complex stress Y(NO) did not show a decreasing trend from 0 to 1 day of treatment, unlike the changes in chemical stress. Y(NO) showed a significant increase from the first day, indicating that the weed initiated a photoprotective mechanism on the first day of complex stress and that more and more light energy was dissipated by thermal diffusion with chlorophyll degradation. The sustained changes in Y(NO) of Erigeron canadensis under compound stress were faster, indicating that the heat diffusion ability of Erigeron canadensis was better than that of Digitaria sanguinalis, which also implied that Erigeron canadensis was more sensitive to glufosinate than Digitaria sanguinalis.

### Comparative analysis of the effects of complex stress and single stress on CF parameters of two weeds

**Figure 11:**
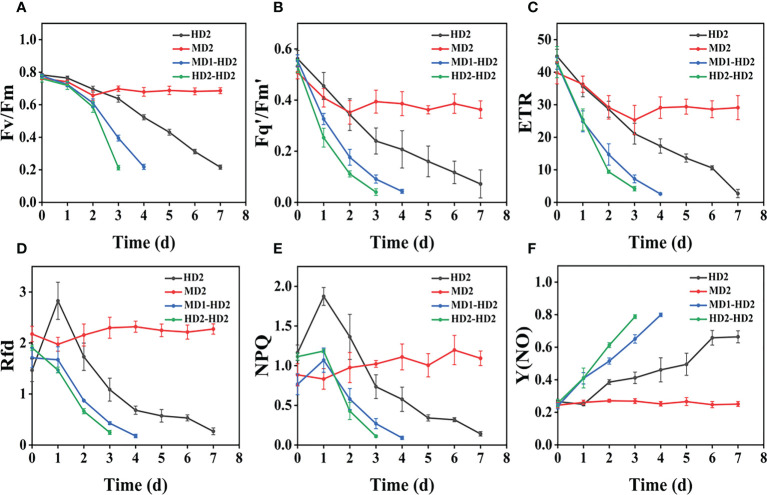
**(A–F)** Variation of each CF parameter with treatment time for different damage modalities (leaf abaxial chemical stress, heavy mechanical stress with light and heavy combined stress) in Erigeron canadensis. Each plot point represents the mean ± SD of three biological replicate determinations. Refer (**Table 1**) for the description of each parameter.

**Figure 12:**
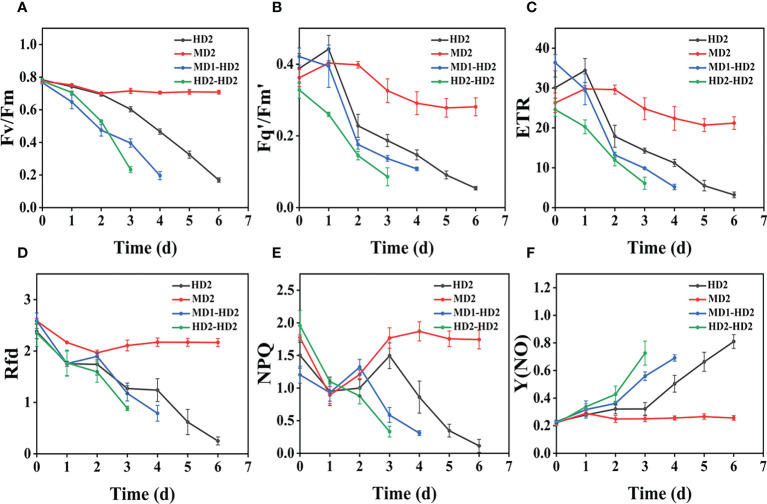
**(A–F)** Variation of each CF parameter with treatment time for different damage modalities (leaf abaxial chemical stress, heavy mechanical stress with light and heavy combined stress) in Digitaria sanguinalis. Each plot point represents the mean ± SD of three biological replicate determinations. Refer (**Table 1**) for the description of each parameter.

**Figure 13:**
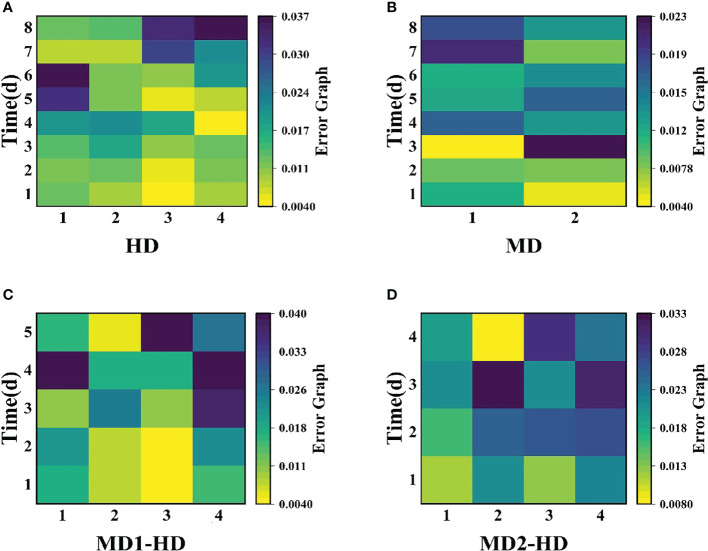
shows the variation of error diagrams of the Erigeron canadensis Fv/Fm at different parts with damage ways. **(A)** the variation of error diagrams of the flying canopy at different parts with chemical damage mode, **(B)** the variation of error diagrams of the Erigeron canadensis at different degrees with mechanical damage mode, **(C)** the variation of error diagrams of the Erigeron canadensis at light mechanical compound damage mode, **(D)** the variation of error diagrams of the Erigeron canadensis at severe mechanical compound damage mode.

Compared with single stress, compound stress not only disrupted the physiological pattern of weeds but also directly damaged the leaf structure of weeds, broke the obstruction of weed cuticle layer, made glufosinate enter into weeds faster, and combined with ATP and occupied the reaction site of GS, the synthesis of GS was blocked, and photosynthesis was affected, which made the weeds die much more efficiently (Ulguim et al., 2019; Takano et al., 2020a; Takano et al., 2020b). It is evident from the time that the composite stress leads to the death of the weed in a far shorter time than when a single stress acts on the weed. As shown in (**Figures 11A–F**, **12A–F**), it was observed from the experimentally obtained curves that the pattern of changes in weeds subjected to complex stress was consistent with that of the single chemical stress, which indicates that chemical stress damages weeds more severely compared to mechanical stress, therefore, compound stress should be dominated by chemical stress and supplemented by mechanical stress, and the heavy mechanical compound stress R increased by 53% compared to chemical stress R. The Fv/Fm parameter is the most intuitive parameter to show the change of photosynthetic rate of the plant, and each parameter was approximately the same for Erigeron canadensis and Digitaria sanguinalis under different stresses. As shown in (**Figures 13A–D**), the error values of Fv/Fm of Erigeron canadensis were larger with longer stress time, indicating that the physiological changes of weeds under stress were stronger with increasing time. We found that in (**Figures 13A–D**), the error values of the back part of the leaves under compound stress, however, all became smaller after 7 days of stress. The reason for this is that the back part of the leaves under compound stress is the most severely damaged part, and the longer the stress time is, the faster its chlorophyll disappears and the smaller its photosynthetic capacity becomes, so that it has almost no photosynthetic capacity, so its error values change less and less.

## Conclusion

This study shows the potential of using CF images to monitor Erigeron canadensis and Digitaria sanguinalis when subjected to abiotic stresses (mechanical, chemical). The response of Erigeron canadensis and Digitaria sanguinalis to abiotic stresses was observed by monitoring the changes in the obtained CF parameters. These changes depended on the type, site and extent of the stressor. The most influential parameters were Fv/Fm, Fq’/Fm’, ETR, Rfd, NPQ, and Y(NO), all of which decreased after the experimental treatment except for Y(NO). Fv/Fm is the most direct window indicating the change in photosynthetic quantum yield, Fv/Fm decreases continuously from the beginning, so chemical stress acts irreversibly on the weed, and chemical stress acts on different parts of the plant, and the maximum photosynthetic inhibition reaches R=75% in the back part of the leaf for Erigeron canadensis and Digitaria sanguinalis. The different levels of mechanical stress, heavy mechanical damage had a greater effect on Erigeron canadensis and Digitaria sanguinalis, with R=11% for heavy mechanical stress, and chemical stress had a significant effect on all parameters of CF compared to mechanical stress, which indicates that chemical stress is more severe than mechanical stress in photoinhibition of photosynthesis. The effect of compound stress on CF parameters was similar to that of chemical stress, but in time it was evident that heavy mechanical compound stress was going to be more severe damage stress on Erigeron canadensis and Digitaria sanguinalis, and the changes in CF parameters were especially obvious, and the compound stress reached 71%-73% of R after only 3-4 days. In summary, Fv/Fm, Fq’/Fm’, ETR, Rfd, NPQ, and Y(NO) can be used as indicator parameters for detecting two abiotic stresses, and usually, the values of all their fluorescence parameters change when affected by stress, and chlorophyll content levels increase or decrease depending on the degree of stress. These results indicate that different types, sites and levels of stress have different effects on photosynthetic activity as well as fluorescence parameters, which will help to inform and guide the flexible design of mechanical weed control, the optimization of chemical weed control on rake spraying methods and the proposal of new weed control models.

## Data availability statement

The raw data supporting the conclusions of this article will be made available by the authors, without undue reservation.

## Author contributions

LQ and KC: Conception, methodology, software, formal analysis, investigation, writing-review and editing. HL: Conception, writing-review and editing, supervision and project management. TBC and WL: Fluorescence processing. TYC, FX, ZL and TG: Data collation, conceptualization and supervision. DS and WJ: Data collation. All authors have read and agreed to the published version of the manuscript.
